# Double strain probiotic effect on *Helicobacter pylori* infection treatment: A double-blinded randomized controlled trial

**DOI:** 10.22088/cjim.8.3.165

**Published:** 2017

**Authors:** Mehdi Haghdoost, Sepehr Taghizadeh, Majid Montazer, Parinaz Poorshahverdi, Ali Ramouz, Sanam Fakour

**Affiliations:** 1 Department of Infectious Diseases, Tabriz University of Medical Sciences, Tabriz, Iran.; 2 Department of Cardiothoracic Surgery, Tabriz University of Medical Sciences, Tabriz, Iran.; 3 Research Fellow, Tabriz University of Medical Sciences, Tabriz, Iran.

**Keywords:** *Helicobacter pylori*, Standard triple therapy, Probiotic, *Lactobacillus*, Recurrence

## Abstract

**Background:**

A decreased rate of successful *helicobacter pylori* (H.pylori) infection treatment has revealed serious demand for more effective regimens to eradicate infection. Therefore, probiotics have recently been considered to increase the rate of antibiotic regimens efficacy in *H. pylori* infections. In current randomized controlled trial, we evaluated the effect of double strain probiotic combination with standard triple therapy (STT), in the eradication rate of *H. pylori* infection.

**Methods:**

In current randomized placebo-control study, all patients (176 subjects) underwent the STT for 10 days. However, the study group received triple therapy for the eradication of *H. pylori* with supplement of *Lactobacillus* probiotic for 4 weeks and placebo was administered to control group, as well. Adverse effects of the antibiotic regimen were recorded for all patients. Six weeks after the cessation of probiotic intake, all patients underwent *H. Pylori* with fecal antigen of test, followed by a recurrence evaluation six months later.

**Results:**

There was no significant difference in demographic data and presenting symptoms between the study groups. The eradication rate of *H. pylori* infection was significantly higher in probiotic group (78.4%), compared to that of placebo group (64.8%) (P=0.033). In addition, adverse events were significantly less prevalent in patients that received probiotic (P=0.047). Nonetheless, there was no significant difference in terms of infection recurrence during a 6-month follow-up (P=0.07).

**Conclusion:**

Double strain probiotic in combination with STT increased the eradication rate of *H. pylori* infection, while the adverse events due to antibiotic therapy decreased.

*Helicobacter pylori* (*H. pylori*) is a gram-negative spiral-shaped bacterium that colonizes the human stomach. It is strongly associated with gastritis and peptic ulcer diseases, while it has been considered as the main cause of primary duodenal ulcers in children. Furthermore, *H. pylori* plays a key role in predisposing of gastric adenocarcinoma and MALT lymphoma (1-5). The prevalence of infection has been estimated to be as high as 90%, whereas the rate of prevalence in developed countries is below 40% (6). The standard eradication therapy of *H. pylori* consists of a proton pump inhibitors (PPIs such as omeprazole, lansoprazole and pantoprazole) and two antibiotics (amoxicillin 1000 mg plus either clarithromycin 500 mg or metronidazole 400 mg) all given twice a day, during a period of 7 to 14 days (7).

During recent years, despite the triple therapy efficacy and high compliance, a constant decline has been reported in eradication rates to less than 55%, subsequent to standard therapy in both adults and pediatrics (8). The falling rate in treatment success is mostly based on bacterial resistance to prescribed antibiotics, yet, antibiotic-associated adverse effects, including diarrhea, nausea, vomiting, abdominal pain, and bloating, decrease the therapy compliance limiting the use of antibiotics (7, 9, 10). Therefore, there is a great interest over the development of a new treatment regimens including administrating of probiotic supplements besides standard protocol to improve eradication rate.

The World Health Organization (WHO) and the Food and Agriculture Organization (FAO) defined probiotics as live microorganisms, conferring a health benefit on the host (11). Although, there are several clinical trials which have been included in reviews and meta-analyses suggesting efficacy of probiotics as an adjuvant treatment to be of several benefits, in improving the treatment tolerability and reducing the side effects of triple therapy. Some reviews reported no significant improvement in *H. pylori* infection treatment, subsequent to of consumption probiotics (12-15). 

Thus, with due attention to current controversies on the effect of probiotics on the eradication of *H. Pylori* infection and reducing the side effects of therapies, we aimed to evaluate the accuracy of probiotics on the eradication of infection, as well as to investigate the efficiency of probiotics in the recurrence rate after 6 months.

## Methods

During a one-year period, between November 2015 to November 2016, a prospective randomized placebo-control trial was conducted in infectious diseases wards of Sina and Imam Reza Hospitals affiliated to Tabriz University of Medical Sciences, Tabriz, Iran, to compare the efficiency of adding probiotics to investigate the modification of *H. pylori*.

Consequently, 176 consecutive patients, aged between 15 and 40 of dyspeptic symptoms and with positive stool antigen test for *H. pylori*, were enrolled in the study. Inclusion criterion was defined as the compliance of patients for therapy by using more than 80% of drugs during the treatment period. The exclusion criteria were: 1) antibiotic treatment in the past, 2) consumption of H_2_-receptor antagonists, bismuth or PPIs 2 weeks prior to study start, 3) *H. pylori* eradication therapy during the past 5 years, 4) the use of NSAIDs and corticosteroids, 5) severe diseases such as malignant disease or history of upper gastrointestinal (GI) problems, such as diaphragmatic hernia, barrette esophagus, and achalasia, 6) pregnancy or lactation, 7) history of immunosuppressive drugs consumption, 8) patients with hereditary immunodeficiency, 9) patients suffering from poorly controlled diabetes, 10) individuals not capable to adhere therapy protocol. All patients provided written informed consent. This study was approved by the Ethics Committee of Tabriz University of Medical Sciences and registered in Iranian Registry of Clinical Trials, (IRCT) (IRCT2016060628304N1).

Randomization was centrally conducted in all groups using standard methodology with a computer-generated list, subsequent to patients’ enrollment. To prevent bias in participant selection and follow-up, we used a combination of both intention-to-treat (ITT) and per protocol method. Prior to drugs administration, all patients presenting symptoms were recorded. Patients received the standard triple therapy (STT) including, pantoprazole (40 mg daily, before breakfast), amoxicillin (1000 mg b.i.d., after meals), clarithromycin (500 mg b.i.d., after meals)) for 10 days. Although, the probiotic group received the triple therapy for the eradication of *H. pylori* with supplement of Prodigest^®^ (500 mg b.i.d.) (Gostaresh Milad Pharmed Co. Tehran, Iran) and the control group received triple therapy with placebo. The Prodigest^® ^capsules contain two bacteria strain including *Lactobacillus* and *Bifidobacterium*, with total viable count (TVC) of 15 × 10^8^ CFU/per capsule. 

The Prodigest^® ^and placebo were continued up to 4 weeks after triple therapy. At least 6 weeks after the cessation of probiotic intake all patients were tested for the eradication by measuring fecal antigen of *H. pylori *(Using TOYO H.PYLORI ANTIGEN Test Stool). Also, six months after cessation of probiotic supplement, *H. pylori* status was rechecked to determine relapse rate, respectively. 

All data were analyzed using SPSS Version 23 Software (SPSS Inc. Chicago, IL). Student’s t-test was used to determine the nonparametric proportions between the two study groups. Moreover, Pearson’s chi-square test and Fisher’s exact test were carried out to determine the efficacy of the two treatments. Furthermore, we used direct-regression test for analyzing the effects of variations on the obtained results. The results with p<0.05 were considered to be statistically significant.

## Results

**Figure 1:**
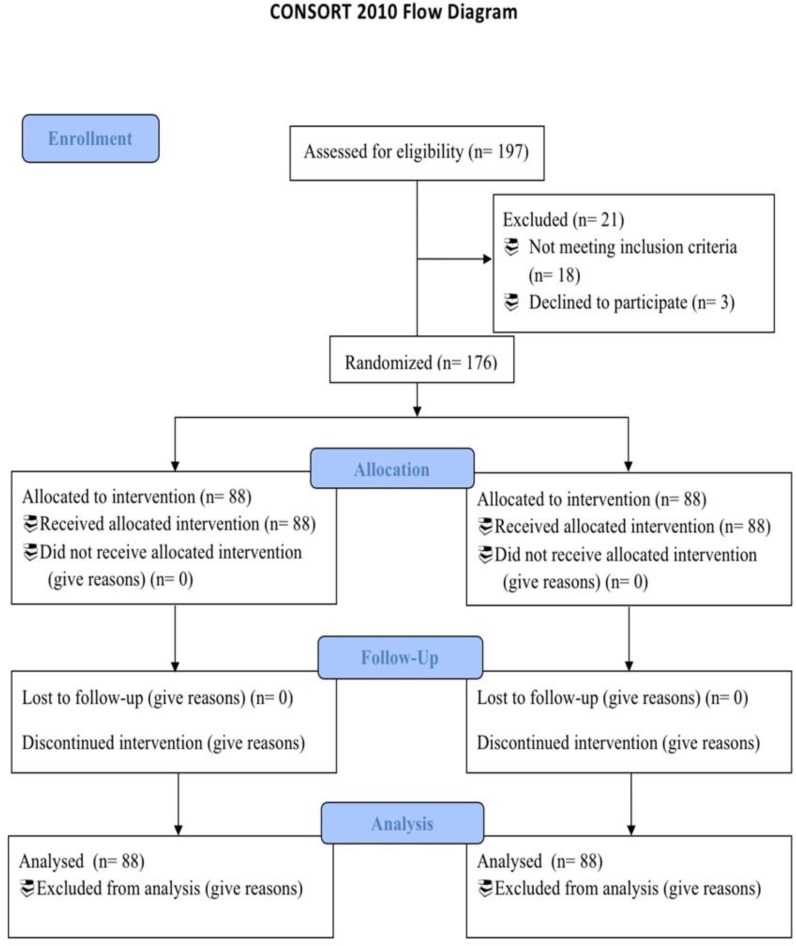
CONSORT diagram detailing the study selection process

In current randomized controlled trial, 176 patients with *H. pylori *infection diagnosis were enrolled and randomly allocated to study (88 patients) and control (88 patients) groups (figure 1). Of these, 95 (53.9%) patients were males, and the mean age was 28.34±5.78 year. Patient demographic data are summarized in table 1, considering the case (A) and control (B) groups. There were no significant differences in terms of age and gender between the study groups. While comparing the primary severity of the symptoms, there was no significant difference in terms of prevalence of presenting symptoms between the study groups (P: 0.073).

Patients underwent *H. pylori *fecal antigen test, in which a month later, it showed positive results in 50 (28.4%) patients. Though, *H. pylori *eradication during a one-month follow-up after treatment was significantly higher in the group that received probiotic supplements (P=0.033). The prevalence of adverse events subsequent to antibiotic consumption including epigastric pain, diarrhea and vomiting is listed in table 1. Although, there was only significant difference in the prevalence of epigastric pain between the study groups (P=0.040), the overall prevalence of adverse effects was significantly lower in patients that received combined STT and probiotic regimen (P=0.047). To evaluate rate of recurrence during a 6-month follow-up, the patients who had positive *H. pylori *antigen in the first month after treatment (19 and 31 patients in groups A and B, respectively), were excluded. Subsequent to patients’ exclusion, we compared age and gender distribution between reformed study groups, which showed no significant difference (table 2.). Fecal antigen test revealed, infection recurrence in 6 (8.6%) patients in group A and 11 (19.2%) patients in group B suffered *H. pylori *at the end of a 6-month follow-up, however, there was no significant difference between the study groups (P=0.070).

Linear regression was used to evaluate the effect of age, gender and treatment protocol on the eradication of infection and rate of treatment success, whereas, treatment protocol was the only variable to significantly affect the successful eradication rate in *H. pylori *infection (P=0.038). 

**Table 1.** Patient demographic data, presenting symptoms and habitual history

**Groups**	**STT (n=88)**	**Probiotic (n=88)**	**P value**	
**Gender (%)**				
Male Female	42 (46) 46 (54)	47 (53.4) 41 (46.6)	0.766	
**Presenting Symptoms (%)**				
Indigestion Heartburn Regurgitation Nausea GI bleeding	25 (28.4) 21 (23.8) 12 (13.6) 6 (6.8) 7 (7.9)	27 (30.6) 26 (29.5) 8 (9) 8 (9) 4 (4.5)	0.434 0.248 0.238 0.391 0.268	0.647
Smoking History (%)	31 (35.2)	28 (31.8)	0.375	

STT: Standard triple therapy

**Table 2:** Eradication rate of *H. pylori* and therapy adverse events in patients

**Groups**	**STT(n=88)**	**Probiotic (n=88)**	**P value**	
Eradication	57 (64.8)	69 (78.4)	0.033	
**Adverse Events**				
Epigastric Pain Vomiting Diarrhea	10 (11.3) 10 (11.3) 3 (3.4)	3 (3.4) 4 (4.5) 7 (7.9)	0.04 0.81 0.165	0.047
Gender*				
Male Female	22 (48.2) 35 (61.4)	28 (40.6) 41 (59.4)	0.483	
Recurrence after 6 months*	11 (19.2)	6 (8.6)	0.07	

* After exclusion of infected patients

## Discussion

*H.pylori *infection is a common condition that imposes financial burdens in health systems, considering its impact on the development of gastric ulcers as well as metaplastic changes (16-20). In late twenties, the SST (including amoxicillin, clarithromycin, and PPIs of the *H. pylori *infection provided the successful treatment rate up to 95% (17, 21, 22). Nevertheless, according to recent studies, increasing antibiotic resistance and treatment side effect leading to poor compliance with therapy has led to reduced rate of infection to less than 75% (23, 24). Consequently, several studies have been conducted to evaluate the efficacy of novel therapies, such as hybrid and concomitant regimen and probiotic compounds including regimen (17, 25, 26).

In current double-blind randomized controlled trial, we compared the efficacy of probiotic compounds on STT for *H. pylori *infection. Our results showed a significant increase in the rate of infection eradication in patients that received probiotic combined with STT comparing with the patients that received STT regimen. Subsequent to consumption of probiotic for 4 weeks, infection eradication increased to 78.4%, compared to 64.8% in STT group. During a 6-month follow-up, although, the patients’ symptoms including dyspepsia and epigastric pain were resolved in both groups, the prevalence of symptoms was significantly lower in the probiotic group. Nonetheless, there was significantly lower prevalence of regimen adverse effects in probiotic groups. Yet, *H. pylori *fecal antigen test revealed no difference in the rate of infection recurrence between the study groups. In a recent systematic review, Zhang et al. have reported probiotics administration without regarding their strain, combined with standard *H. pylori *treatment, which has led to approximately 10% increase in the rate of *H. pylori *infection, as well as reduction in the prevalence of adverse events (23). 

Similarly, McFarland et al. reviewed 19 randomized controlled trials and reported significant improvement in terms of *H. pylori *eradication and adverse events subsequent to combined administration of eradication therapy and multi-strain probiotics (27). However, some meta-analyses suggested no significant difference between STT regimen alone and STT regimen combined with probiotics, in the rate of eradication in *H. pylori *infection, but the strain of probiotics and administration dosage has not been taken into consideration (28, 29).

There are several studies discussing the efficacy of single strain probiotics on therapeutic outcomes of the STT in the eradication of *H. pylori *(30, 31). In a review, the administration of *Lactobacillus* containing probiotics found to raise *H. pylori *eradication rate more than multi-strain probiotics (32, 33). While the results of recent studies including a randomized placebo-controlled study have shown an inhibitory effect of single strain (*Lactobacillus reuteri*) probiotic on *H. pylori *growth, as well as a 9.1% decrease in the rate of antibiotic related adverse effects during the standard regimen prescription (34, 35). In the current study, we found that STT in combination with *Lactobacillus* and *bifidobacterium *containing probiotic was significantly helpful, not only in increasing the rate of *H. pylori *eradication, but also, in reducing STT regimen administration adverse events, which was consistent with previous study results. We found no significant effect of probiotic administration on infection recurrence after 6 months. To our knowledge, there are few studies that evaluated the efficacy of probiotics combined with conventional STT regimen in the treatment of *H. pylori *infection in Iran. In a randomized trial, *Saccharomyces boulardiito* that contained probiotics improved the incidence of STT adverse events, but with no significant effect on *H. pylori *eradication rate (20). Shavakhi et al. discussed the effect of adding combined quadruple (PPI, bismuth citrate, amoxicillin and clarithromycin) and multi-strain probiotic regimen (36). Nonetheless, they report no significant difference in the treatment of *H. pylori *infection by adding probiotic compounds to quadruple regimen. In the present study, we administered standard triple therapy for 10 days and the study group received probiotic compound containing *Lactobacillus* strains for 28 days. The results showed a significant higher eradication rate for this regimen as compared to probiotic regimen in Shavakhi’s study. 

To compare our results with previous studies, it can be concluded that double-strain probiotics are more effective than multi-strain probiotic in improving the rate of *H. pylori* infection eradication. Neverthess, in a recent review, McFarland et al. have reported that few probiotic strains including *Lactobacillus* may be effective in the improvement of *H. pylori* infection eradication and reduction of adverse events (27). As a result, they suggested probiotic strain type as the most effective factor in predicting the efficacy of regimen.

Some studies have suggested that the efficacy of probiotic compounds may depend on several factors, such as dosage and its components (36). Though considering the abovementioned studies and our findings, we hypothesized that double-strain probiotics seem to be more effective than multi-strain probiotics, not only in enhancing the rate of *H. pylori* eradication, but also in terms of adverse events. Although, we detected no significant difference between probiotic containing regimen and STT in the rate of infection recurrence, probiotics role should be evaluated in further studies. In the current study, fastidious study design, scrupulous patient enrolment and careful follow-up were employed to evaluate single strain probiotic effect on the improvement of STT regimen efficacy. In spite of that, our study had some weaknesses and limitations, which as follows: first, due to lack of standardized protocol for probiotic supplementation, administration performed based on previous study methods, second, our patients received STT, despite that, bismuth quadruple therapy has been suggested by the Iranian Association of Gastroenterology and Hepatology (IAGH) to be more effective in Iranian population. In conclusion Administration of *Lactobacillus* probiotics combined with standard triple therapy increased the rate of *H. pylori* infection eradication to 78%. Since probiotic consumption reduces adverse events due to antibiotic regimen, it enhances therapy compliance significantly. However, even though it revealed a lower rate of infection recurrence at the end of six-months follow-up, it was not statistically significant.
